# Process optimization of ultrasound-assisted treatment for soya bean protein isolate/polyacrylamide composite film

**DOI:** 10.1098/rsos.180213

**Published:** 2018-07-04

**Authors:** Xinwei Cao, Bo Zhu, Yichuan Gao, Jianli Liu, Weidong Gao, Xiaoling Gai, Wei Bao

**Affiliations:** 1 Key Laboratory of Eco-textiles, Ministry of Education, Jiangnan University, Wuxi 214122, People's Republic of China; 2 Beijing Key Laboratory of Environment Noise and Vibration, Beijing Municipal Institute of Labor Protection, Beijing 100054, People's Republic of China; 3 Wuxi Customs, Wuxi 214001, People's Republic of China; 4 Wuxi Entry-Exit Inspection and Quarantine Bureau, Wuxi 214101, People's Republic of China

**Keywords:** ultrasonic treatment, soya bean protein isolate, polyacrylamide, tensile strength, process optimization, response surface method

## Abstract

In this paper, composite films composed of soya bean protein isolate (SPI) and polyacrylamide (PAM) were prepared under variations of ultrasonic power, treatment time and heating temperature. The effects of the major processing parameters related to ultrasonic-assisted treatment were evaluated and optimized through the single-factor analysis and Box–Behnken design (BBD), respectively, when the tensile strength of composite films was considered as the response value. The single-factor analysis was carried out to study the effects of ultrasonic power, treatment time and heating temperature on the viscosity and cohesion of the slurry and the tensile strength of SPI/PAM composite films, which also provided a reasonable data range of each factor for further optimization. Experiment results indicated that these three factors play a significant role in the tensile strength of films. Then BBD was applied to optimize the treatment conditions of these three factors, using the tensile strength of films as the response value. According to the interactive second-order polynomial model of three factors and the three-dimensional response surface, the maximum tensile strength of films was obtained under the optimal condition. To verify the reliability of the model, the experiment with the optimal condition was conducted, and results demonstrated that the observed tensile strength was in agreement with the predicated one. Also, the morphology and water solubility of the films showed that the film can be coated on the yarns evenly and removed clearly.

## 1. Introduction

Warp sizing, a key process in textile production, imparts protection to yarns to withstand friction during weaving, and thus determines the qualities of textiles and the efficiency of textile weaving processes [1]. However, the textile industry, especially desizing, consumes large volume of water and chemicals, which makes the industry a major contributor to water pollution across the world [2]. Soya bean protein isolate (SPI), extracted from soy meal and containing more than 90% protein, is abundant, cost-effective and biodegradable [3]. It is made up of 35% conglycinin (7S) and 52% glycinin (11S) [4]. In previous studies, SPI has been reported to have good film forming properties owing to the formation of disulfide linkages of 11S protein and the potential of being textile sizing agents [5]. However, without the modification of physical, chemical or enzymatic treatment, its film is rather brittle, and has relatively poor mechanical properties [6].

Many studies have been done to improve the flexibility of SPI films because flexibility plays an important role in weaving sizing. The processing conditions include pH, temperature, heating time, pressure and plasticizers [7]. Mauri & Añón [8] have found that the intensity of protein film networks maintained by covalent and non-covalent bonds depended on the pH of the initial solution. Apart from pH effect, heat and pressure also affected the grade of denaturation of SPI. The flexibility and integrity of protein network was enhanced owing to the exposure of hydrophobic, thiol and other active groups in SPI after heating [9,10]. Furthermore, additives can strongly improve the mechanical properties of SPI films, small molecules containing amino, imino groups or hydroxyl groups have been reported as good plasticizers because of their ability to reduce intermolecular hydrogen bonding to increase intermolecular spacing [11,12]. Glycerol is the most widely used plasticizer in SPI films because of its small size and hydrophilic nature, which makes it compatible with SPI films [13]. However, not much has been discussed regarding the properties of the film combining SPI with polyacrylamide (PAM) which is widely used as a sizing agent in weaving sizing.

It is necessary to develop a novel technique for enhancing the mechanical properties of SPI films. Ultrasonic treatment consists of a series of longitudinal waves with differences in density, and spreads around the medium [14]. The ultrasonic-assisted method [15,16] is a competitor and popular technique owing to its advantages over other conventional techniques, such as short treatment time, user-friendliness and low energy consumption. In recent years, the power ultrasound was widely introduced to improve the functional properties of proteins. Ultrasonic treatment resulted in partial unfolding, the breaking of peptide bonds and reduction of intermolecular interactions, leading to the changes in protein functional properties [17,18].

The objective of the present work was to contribute to a thorough study of ultrasound-assisted treatment for SPI/PAM composite films. The single factor experimental design was used to provide a reasonable data range of each factor as ultrasonic power, treatment time and heating temperature for the further optimization study. The Box–Behnken design (BBD) response surface method was adopted to determine the optimal condition for achieving the better mechanical properties of SPI/PAM films.

## 2. Material and methods

### 2.1. Materials

SPI (protein content, 94.4% on dry basis, as determined by Kjeldahl determination, and the test report was provided by Qingdao F&C Testing and Analysing Co., Ltd) was purchased in Linyi pine Biological Products Co., Ltd (Shandong, China). Sodium hydroxide (NaOH) and glycerine were purchased from Sino pharm Chemical Reagent Co., Ltd. PAM was purchased by Feng yuan Co., Ltd (Changzhou, China).

### 2.2. Methods

#### 2.2.1. Preparation of soy protein isolate/polyacrylamide composite film

According to the method of Wang *et al.* [19] proposed with slight modification, the SPI and PAM solutions were produced by using distilled water as the solvent. Modified soy protein sizes were prepared by stirring 6 wt% SPI solutions which contained 1 wt% sodium hydroxide and 1.8 wt% glycerine at 200 r.p.m. (RW20, IKA, German) for 5 min at ambient temperatures. Then 6 wt% PAM solutions were added to the soy protein sizes to make sure that the ratio of two sizes was 40/60. Then the mixture slurry was placed and fixed in an ultrasonic process (XO-SM200, ATPIO Instrument Manufacturing Co., Ltd, Nanjing, China). This was followed by heating for 30 min at a set temperature. Then the slurry was cast onto a Teflon plate for drying at 20°C and 65% humidity, which was placed into a temperature and humidity chamber (Shanghai Yiheng Scientific Instrument Co., Ltd, China) for about 35–40 h.

#### 2.2.2. Intrinsic viscosity of sizing solution

After the modified treatment using ultrasonication, viscosity of the sizing solution with a solution concentration of 6 wt% was measured with the SNB-1 viscometer (Shanghai Fangrui Instrument Co., Ltd, China). During the experiment, all measurements in terms of mPa.s were carried out at the treatment temperature.

#### 2.2.3. Cohesion of fibres

The tensile properties of rovings will be used to evaluate the cohesion ability of fibres of the prepared sizing solution, in which the rovings will be soaked for 5 min. In rovings, the fibres are loose, aligned and can be easily drawn out. So, the increase in the strength of rovings can be used as a good indicator of the cohesion among fibres [20–22]. The rovings wound on frames were immersed in the sizing solution after the modified treatment with the concentration of 1 wt% for 5 min and then were dried in an oven. Tensile properties of rovings were tested on a Shimadzu tensile tester AGS-X (Shimadzu Corporation, Japan) using a gauge length of 10 cm and a crosshead speed of 50 mm min^−1^. At least 20 samples of each condition were tested, the average and standard deviation were reported.

#### 2.2.4. Tensile strength measurement

Firstly, the thickness of film was measured by randomly selecting five points of each sample and the average values were calculated (the general thickness was 0.03–0.06 mm). Then films formed were tested for breaking force on a Shimadzu tensile tester AGS-X according to the ASTM standard D822 [23]. Film samples with the size of 10 × 1 cm were prepared and tested using a gauge length of 10 cm and a crosshead speed of 50 mm min^−1^. At least 20 samples were tested and the average breaking force was reported. The tensile strength of the tested samples was calculated using equation (2.1). It must further be noted that the breaking force and thickness refer to the average ones of the test set:

2.1 $$\text{tensile strength (MPa)}=\frac{\text{breaking force}}{\text{thickness}\times \text{width}}.$$

#### 2.2.5. Experimental design

In our research, a single-factor experiment will be carried out to determine the effects of ultrasonic power, treatment time and heating temperature on the tensile strength of SPI/PAM films. A comparably reasonable data range of each factor can be achieved after the single-factor experiment, which will be used for the further selection of the optimal one. Furthermore, the BBD was applied to determine the optimal condition for the maximum tensile strength of SPI/PAM films in theory. Design Expert software (version 8.0.6) was used for experimental design, analysis and data processing. Each determination in the present experiment was run in triplicate to verify the reliability of the results.

## 3. Results and discussion

### 3.1. Single-factor analysis

#### 3.1.1. Ultrasonic power

**Figure 1.**
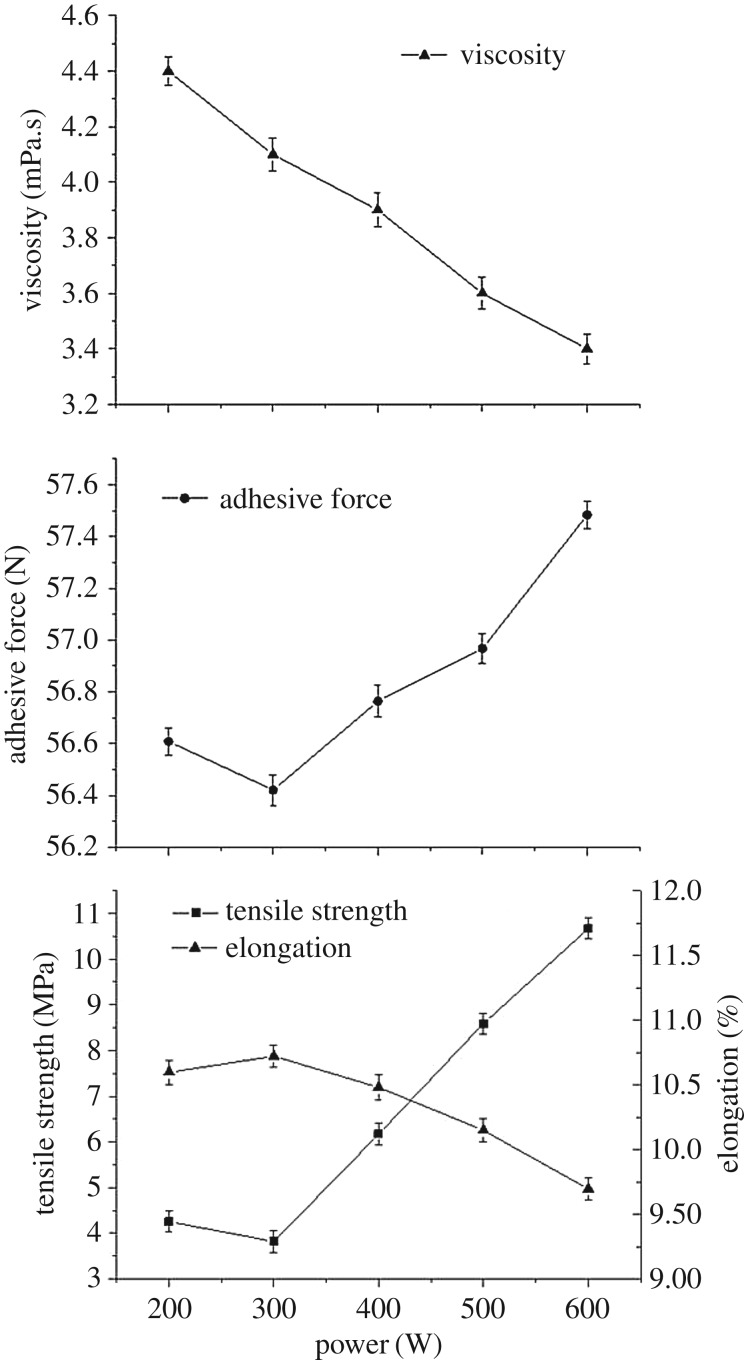
Viscosity and cohesion force of the slurry and the mechanical property of SPI/PAM films at different ultrasonic power.

The mixture of modified SPI and PAM that will be used as sizing slurry was sonicated with five different ultrasonic powers, 200, 300, 400, 500 and 600 W before being heated at 95°C. The treatment time of ultrasound and heat are both 30 min. Considering the mixture of modified SPI and PAM is processed with five different ultrasonic powers, we will get five samples. The viscosity and cohesion of the five slurry samples and mechanical property of the corresponding SPI/PAM films are shown in figure 1.

It is observed that the viscosity of the mixture slurry decreased from 4.4 to 3.4 mPa.s when the ultrasonic power increased from 200 to 600 W. This decrease could be attributed to the denaturation followed by rearrangements and unfolding of molecules occurred to some extent with an increase in sonication power [24]. When ultrasonic power increased from 200 to 300 W, the cohesion of the sizing solution evaluated in terms of adhesive force of rovings and the tensile strength of films both significantly decreased but the flexibility increased correspondingly, which could be explained by the fact that the shear forces produced by ultrasonic cavitation help to reduce intermolecular cohesion by the disruption of hydrogen bonding, as well as hydrophobic and electrostatic interactions [25]. When the power increased from 300 to 600 W, the cohesion of slurry remarkably increased, similarly to the tensile strength of SPI/PAM composite films, and the elongation inversely decreased. Sonication may lead to the formation of aggregates, which could influence the film network structure and mechanical properties on increase in ultrasonic intensity [26]. Furthermore, the increase in ultrasonic energy would increase the motion of molecules and the space between the units of PAM thus favours the movement of polymer chains, which contributed to the increase in permeability properties that have a great impact on cohesion of the sizing solution [27]. On the other hand, in the hybrid system, there are two different interfacial interactions, one is the interaction between the protein and PAM and the other is the interaction between molecule chains of protein or PAM. The two types of interactions resulting from the non-covalent interactions such as hydrogen bonds and electrostatic forces could keep a new balance system after ultrasound irradiation, which plays an important role in the network structure of the hybrid films that influenced the mechanical properties of composite films [28].

#### 3.1.2. Treatment time

**Figure 2.**
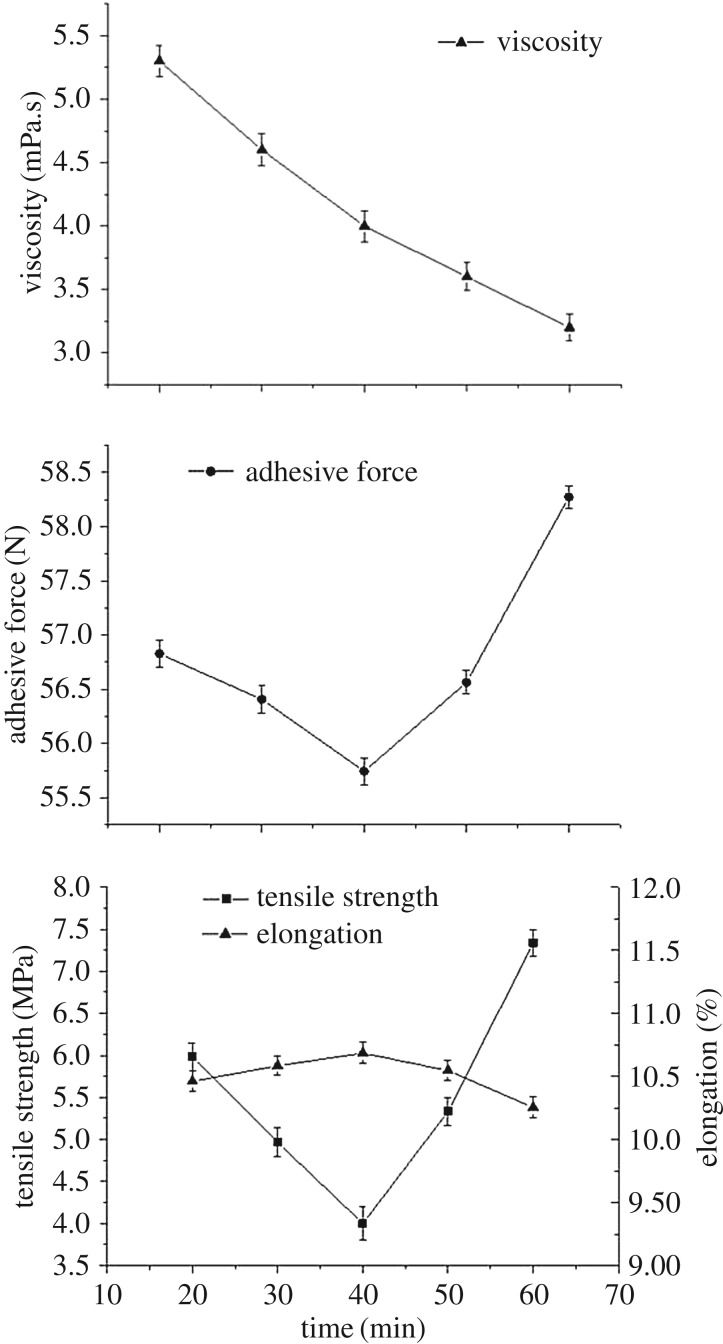
Viscosity and cohesion of slurry and the mechanical property of SPI/PAM films at different treatment times.

The modified SPI and PAM mixture solution was sonicated for 20, 30, 40, 50 and 60 min, with an ultrasonic power of 200 W and then heated at 95°C for 30 min. The viscosity and cohesion of slurry and mechanical property of SPI/PAM composite films at different treatment times are shown in figure 2.

As shown in figure 2, the viscosity of the mixture slurry sharply decreased with the increase of ultrasound treatment time. The viscosity of solutions was determined by the extent of entanglement and intermolecular interactions among polymer molecules which was decreased with ultrasound treatment by weakening of the hydrogen bond and other non-covalent bonds between SPI and PAM molecules [29]. Furthermore, the changes of particle size distribution and decrease in SPI particle size after ultrasound treatment may contribute to the decrease in the viscosity of solutions.

The trend of cohesion of sizing solutions is consistent with the tensile strength of SPI/PAM composite films, which decreases when the treatment time ranged from 20 to 40 min and increased from 40 to 60 min contrary to the elongation of the film. At the early period of ultrasound treatment (during 20–40 min), molecules become more liberated and stretched owing to the cavitation and microstreaming resulted from the sonication. Some of the hydrophobic regions of SPI may be exposed, which previously were located buried within the interior of the molecules [30]. This may be the reason for the decrease in adhesion to cotton rovings that were hydrophilic fibres. Owing to the unfolding and disruption of intramolecular bonds, the compact reticulate structure of SPI was destroyed, which caused the reduction in the tensile strength of the films and the increase in flexibility. Another reason may be the destruction of the crystalline structure of PAM resulting from the cavitational forces of ultrasonic treatment [27]. With the increase of treatment time (40–60 min), hydroxyl radicals of unfolding protein chains promoted the water hydrolysis and radical chain reaction leading to polymerization, resulting in a more compact and homogeneous network [31]. This possibly is the reason for the reduction in the extensibility and the increase in cohesion of the sizing solution and tensile strength of films. Otherwise, the fluidity of the sizing solution increases because of the decrease in viscosity, which is beneficial to the spreading of the fibre to keep close contact with the solution and contributed to a high adhesive force.

#### 3.1.3. Heating temperature

**Figure 3.**
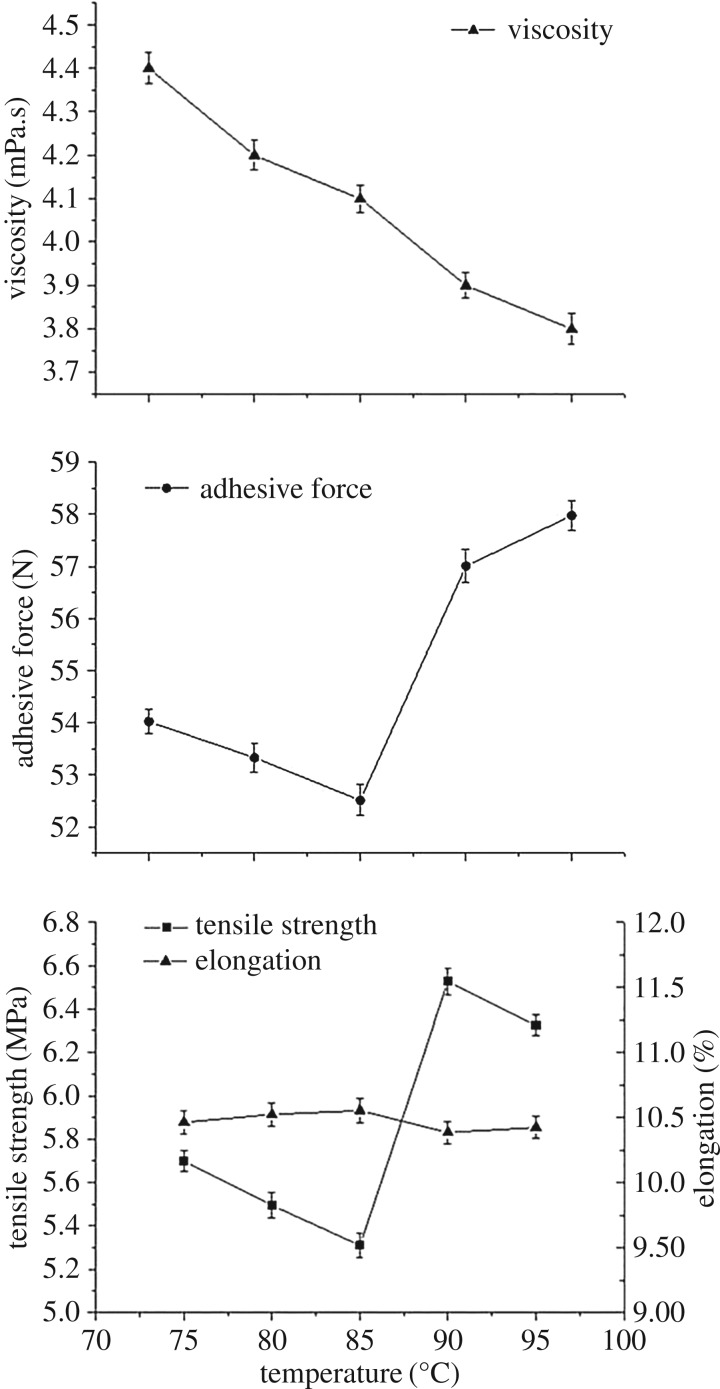
Viscosity and cohesion of slurry and the mechanical property of SPI/PAM films at different heating temperatures.

The modified SPI and PAM sizing slurry was sonicated with an ultrasonic power of 400 W for 30 min and then heated at 75°C, 80°C, 85°C, 90°C and 95°C after the ultrasound treatment. The viscosity and cohesion of slurry and mechanical properties of the SPI/PAM films at different heating temperatures are shown in figure 3.

As shown in figure 3, the viscosity of the solution decreased from 4.4 to 3.8 mPa.s with the increase in treating temperature from 75 to 95°C. Heating treatment changes soya bean protein from its native state to a denatured one, accompanied by an increase in glycinin molecular motion and protein unfolding, disruption of the intramolecular bonding and a decrease in the α-helical and β-sheet structures at the expense of the random coil structure, resulting in extensive denaturation of the secondary structure of soy 11S [32]. Otherwise, the viscosity decreased probably owing to the aggravation of thermal molecular motion with the increase in the heating temperature that weakens the intermolecular forces.

Figure 3 reveals that the cohesion of the sizing solution decreased observably when the heating temperature increased from 75 to 85°C, and increased sharply as the temperature reached 90°C, which was similar to the tendency of the tensile strength of the composite films and contrary to the elongation. When the heating temperature rose from 90 to 95°C, the cohesion of slurry and flexibility increased opposite to the tensile strength of the films. During the early stage of heat treatment (from 75 to 85°C), the denaturation temperature of 7S and 11S globulins, parts of the three-dimensional structure of 11S were unfolded, and parts of the hydrophobic groups which were buried inside before heating, were exposed to water, which can explain the increase in extensibility and decrease in the cohesion and tensile strength from 75 to 85°C. With the continuous increase in heating temperature (from 85 to 90°C), 7S and 11S globulins were denatured completely, the molecular distances were close enough to each other, and intermolecular polymerization occurs through molecular forces and non-covalent bonds. The structural change of SPI results in the formation of aggregates contributing to the compact network, which is responsible for the rapid increase in the tensile strength and cohesion [6]. When the heating temperature reached 95°C, some aggregates might be dissociated into 11S polypeptides that contributed to the decrease in the tensile strength of films and the increase in the elongation [33]. On the contrary, cohesion of the solution increased because the improvement of solubility of the protein enhanced the ability to disperse the protein in water and the interaction of apolar and polar groups with the rovings [34].

### 3.2. Box–Behnken analysis

The BBD experiments were carried out to yield maximum information about factors of ultrasonic power X1, treatment time X2 and heating temperature X3. The interactions of their three factors with minimum experiments will be used to optimize the experimental conditions. A three-factor and three-level factorial BBD was employed with a serial of experiments consisting 17 runs to investigate the effects of factors on tensile strength of SPI/PAM composite films which plays an essential role in warp sizing. Factors and levels of BBD experiment are given in table 1. Design table and results of the BBD experiment are shown in table 2.

**Table 1.** Factors and levels of Box–Behnken design experiment.

		code level		
codes	factors	−1.00	0.00	1.00
X1	ultrasonic power (W)	500	550	600
X2	treatment time (min)	50	55	60
X3	heating temperature (°C)	85	90	95

**Table 2.** Design table and results of Box–Behnken design experiment.

run	X1	X2	X3	Y1 tensile strength (MPa)
1	550.00	55.00	90.00	12.6110
2	500.00	55.00	85.00	7.2061
3	550.00	55.00	90.00	12.3251
4	550.00	50.00	95.00	9.2584
5	550.00	50.00	85.00	7.5807
6	600.00	55.00	85.00	10.1325
7	550.00	55.00	90.00	12.2300
8	600.00	60.00	90.00	12.9305
9	600.00	55.00	95.00	12.7338
10	550.00	60.00	95.00	11.7941
11	600.00	50.00	90.00	10.7117
12	550.00	60.00	85.00	8.1030
13	500.00	55.00	95.00	8.8652
14	500.00	60.00	90.00	9.4789
15	500.00	50.00	90.00	8.4784
16	550.00	55.00	90.00	12.4723
17	550.00	55.00	90.00	12.1293

Through BBD analysis, an interactive second-order polynomial model was used to evaluate the response factor. The linear relationship of response, main variables and their interactions can be formulated as the following equation:

3.1 $$\begin{array}{rl} \text{Y1} & =\text{12.35}+\text{1.56X1}+\text{078X2}+\text{1.20X3}+\text{0.30X1X2}+\text{0.24X1X3}+\text{0.50X2X3}\\ & \;-\text{0.70}{\left(\text{X}1\right)}^{2}-\text{1.25}{\left(\text{X}2\right)}^{2}-\text{1.92}{\left(\text{X}3\right)}^{2}, \end{array}$$

where Y1 is the value of tensile strength of SPI/PAM composite films, and X1, X2 and X3 are independent factors as shown in table 1. Equation (3.1) represented the quantitative effect of factors (X1, X2 and X3) and the interactions on the tensile strength of films. The factors of X1, X2 and X3 were substituted in the equation to obtain the theoretical values of Y1. The analysis of variance (ANOVA) of equation (3.1) is given in table 3.

**Table 3.** Analysis of variance (ANOVA) table for response surface quadratic model by Box–Behnken experimental design. (*R*^2^ = 0.9929, *R*^2^(adj) = 0.9838.)

source	sum of squares	d.f.	mean square	*F*-value	*p*-value
model	64.06	9	7.12	108.89	<0.0001^a^
X1	19.47	1	19.47	297.81	<0.0001^a^
X2	4.93	1	4.93	75.35	<0.0001^a^
X3	11.59	1	11.59	177.30	<0.0001^a^
X1X2	0.37	1	0.37	5.68	0.0487^a^
X1X3	0.22	1	0.22	3.39	0.1079
X2X3	1.01	1	1.01	15.50	0.0056^a^
(X1)^2^	2.07	1	2.07	31.71	0.0008^a^
(X2)^2^	6.60	1	6.60	100.96	<0.0001^a^
(X3)^2^	15.48	1	15.48	236.81	<0.0001^a^
residual	0.46	7	0.065		
lack of fit	0.31	3	0.10	2.82	0.1708
pure error	0.15	4	0.037		
cor total	64.52	16			

^a^Significant at 95% confidence degree (*p* < 0.05).

According to the results of the regression and ANOVA analyses on the model of the BBD, the predicted values obtained from the quadratic model have a good agreement with the actual values, as the correlation coefficient (*R*^2^) was 0.9929, which is close to 1. Besides, the value of the adjusted *R*^2^ was 0.9838, which indicated that the model was highly significant and can be used to navigate a better precision and reliability for the experiments. The data in table 3 showed that the model *F*-value was 108.89 and *p*-value was less than 0.0001, which implied that the regression model was acceptable with the variance analysis at the 95% confidence limit. Additionally, all variables and interacting factors were found to be significant (*p* < 0.05) except for X1X3, when the calculated *p*-values were used as evaluation parameters. The *p*-value of lack-of-fit was larger than 0.1, which indicated that the model fit well the experimental data and could explain the variability in the response.

**Figure 4.**
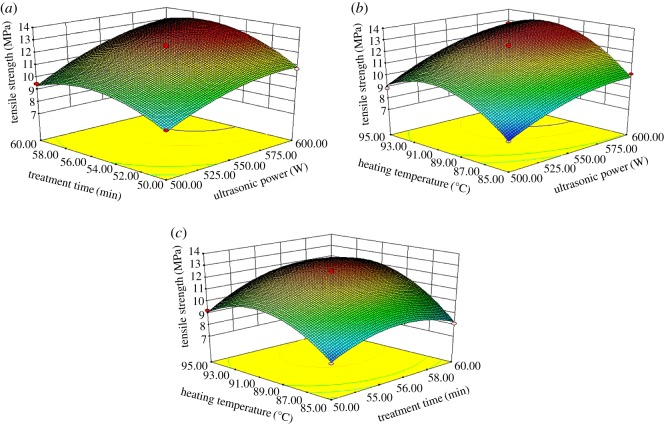
The three-dimensional response surface for the tensile strength of prepared films. (*a*) Effects of ultrasonic power and treatment time on the tensile strength of films; (*b*) effects of ultrasonic power and heating temperature on the tensile strength of films; and (*c*) effects of treatment time and heating temperature on the tensile strength of films.

Furthermore, the three-dimensional response surface for optimization of factors of ultrasound-assisted treatment for SPI and PAM slurry could further and directly interpret the effects of each two independent variables and their interactions on tensile strength of films. The three-dimensional response surface illustrations for tensile strength of films are presented in figure 4.

Figure 4*a*,*b* shows that the tensile strength (Y1) firstly increased and then declined slowly when ultrasonic power (X1) increased from 500 to 600 W, and treatment time (X2) and heating temperature (X3) were set at a certain value. This was mainly attributed to the fact that the formation of soluble protein aggregates and then the structure of film network becomes more compact and uniform when the ultrasonic power increased from 500 to 575 W. The improvement of the tensile strength of films was no doubt a necessary consequence of the increase in ultrasonic power. However, the extra ultrasonic energy may destroy the structure of protein by dispersing the insoluble protein aggregation because of the cavitation and microstreaming of sonication, which will cause a decrease in tensile strength of films.

Figure 4*a*,*c* demonstrates that the value of Y1 first increased and then decreased when treatment time (X2) ranged from 50 to 60 min, X1 and X3 were constant, which was in agreement with the tendency of ultrasonic power. High-frequency oscillation of ultrasound will make the protein more homogenized, through accelerating the formation of soluble aggregates with the association of unstable aggregates [35]. However, the process is reversible. Longer treatment time will contribute to the formation of unstable aggregates from soluble aggregates, which will destroy the steady structure and decrease the tensile strength of films.

In figure 4*b*,*c*, Y1 has a trend of increasing first and decreasing followed while the level of X3 (heating temperature) ranged from 85 to 95°C. High temperature could promote the denaturation of protein, and result in the formation of aggregates through molecular forces and non-covalent bonds, which makes the increase in the tensile strength of films. However, the continuous heating will cause the completed denaturation of protein, and some aggregates might dissociate into 11S polypeptides, which will contribute to the decrease in the tensile strength of films.

### 3.3. Method validation

To verify the validity of the response surface analysis method, the experiments with the optimum conditions: ultrasonic power of 580 W, treatment time of 57 min and heating temperature of 93°C (taking practical operability into consideration) were conducted. Then the properties of the composite films, in addition to the tensile strength, the morphology and solubility, were tested.

#### 3.3.1. Tensile strength

The predicted and experimental response value, given the optimal parameters after optimization with response surface method, are shown in table 4. The predicted maximum tensile strength of films was 13.4075 MPa, and the actual value was 13.2948 MPa, which is reasonably in good agreement with the theoretical values. This indicated that the optimal experiment parameters deduced by the response surface analysis method were reliable.

**Table 4.** Predicted and experimental response values led by optimal parameters after optimization with response surface method.

			tensile strength (MPa)	
ultrasonic power (W)	treatment time (min)	heating temperature (°C)	predicted	experimental
577.78 (580)	57.22 (57)	93.11 (93)	13.4075	13.2948

#### 3.3.2. Morphology

**Figure 5.**
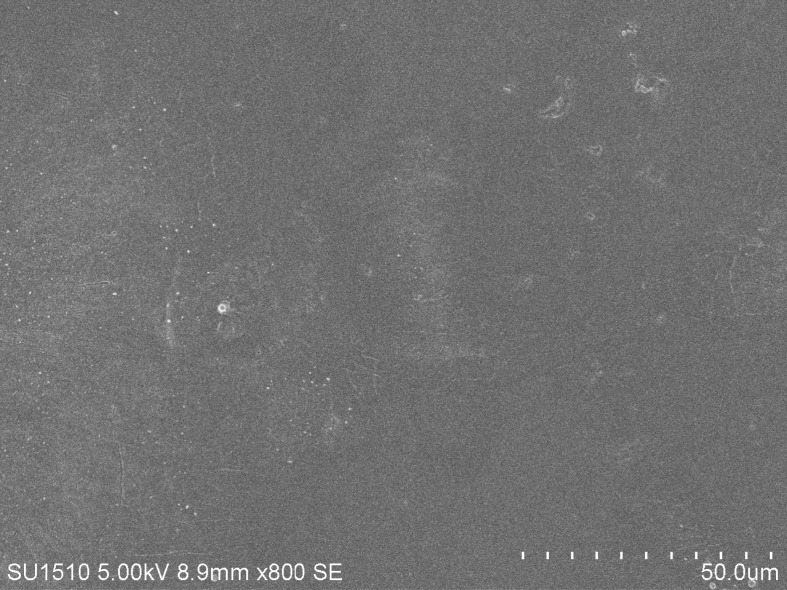
The scanning electron micrographs of SPI/PAM composite films at the optimum conditions.

The morphology of SPI/PAM composite film was determined via scanning electron microscope (SU1510, Hitachi, Japan), which is an informative technique for the detailed examination of surfaces at molecular level. Figure 5 shows that the surface of the film is smooth, compact and continuous without cracks and pores, which indicated that the SPI and PAM mixed up completely and the film can be coated on the yarn evenly to increase the mechanical properties of yarns after sizing.

#### 3.3.3. Water solubility

To study the solubility of the composite films which can significantly affect the desizing process, the breaking time of the size of 10 × 2 cm film samples with a straight line along the length in a 80°C water bath was recorded. During the experiment, it is important to make sure the liquid level coincides with the straight line. The results showed that the breaking time of the SPI/PAM composite films was 10.72 s, while the same blend ratio of oxidized starch and PAM composite films which was widely used in the sizing process was 19.28 s. So, the water solubility of SPI/PAM composite films was highly improved because the shorter the breaking time, the better the water solubility, which indicated that the sizes can be removed from the yarn easily. With the modification of SPI, the solubility of the protein was remarkably improved, which can be a possible reason for the high water solubility of composite film.

## 4. Conclusion

This paper used the single-factor analysis to investigate the impact of ultrasonic power, treatment time and heating temperature on the results of ultrasonic-assisted treatment for an SPI and PAM solution. The results revealed that these three factors have a great effect on the tensile strength of SPI/PAM composite films. Then the BBD response surface method was adopted to optimize experimental conditions by using the tensile strength of SPI/PAM composite films as the response value. When the values were set as 580 W of ultrasonic power, 57 min of treatment time and 93°C heating temperature, the tensile strength of films reached the maximum of 13.2948 MPa. The observed values (13.2948 MPa) were in reasonably good agreement with the predicated values (13.4075 MPa) and the deviation was less than 1%, which indicated that the optimization results were reliable. Also, according to the morphology and water solubility of the film at the optimal conditions, the film can be coated on the yarns evenly and removed clearly. As a result, the ultrasonic-assisted method proposed in this paper can greatly improve the mechanical properties of SPI/PAM films.

## Data accessibility

This article does not contain any additional data.
